# Variations in the composition and frequency of celiac disease epitopes among synthetic wheat lines

**DOI:** 10.3389/fpls.2024.1517821

**Published:** 2025-01-27

**Authors:** Rahman Ebrahimzadegan, Ghader Mirzaghaderi

**Affiliations:** Department of Plant Production and Genetics, Faculty of Agriculture, University of Kurdistan, Sanandaj, Iran

**Keywords:** synthetic wheats, D-subgenome, celiac disease, amplicon sequencing, alphagliadin, immunogenic peptides

## Abstract

Bread wheat serves as an important staple crop in the human diet, largely because of the physicochemical properties of its dough and its protein content. Gluten is the main and complex component of wheat proteins. Despite the significant importance in breadmaking properties, wheat gluten contains some immunogenic peptides capable of triggering a T cell reaction in celiac disease (CD) patients, leading to inflammation in the small intestine. Among gluten proteins, α-gliadins are the most immunogenic components because they possess the primary T-cell stimulating epitopes (DQ2.5-Glia-α1, DQ2.5-Glia-α2, and DQ2.5-Glia-α3), which are primarily located on the D subgenome. Developing new wheat varieties by integrating the D subgenome from various sources is not only useful for introducing low immunogenic gluten, but it can also circumvent the challenging policies arising from the manipulation of wheat genome through transgenic approaches. Here, we performed RNA amplicon sequencing of the most toxic region of alpha-gliadins to analyze the content and composition of CD-related alpha-gliadin epitopes across eight synthetic wheat lines developed from crosses between durum wheat and different Aegilops species containing the D-genome (*Ae. tauschii*, *Ae. crassa*, and *Ae. ventricosa*). By searching the previously identified 121 epitopes and those with one mismatch in our amplicons, we found 54 different α-gliadins epitopes across our genotypes, four of which were new variants. The canonical epitopes were present in all lines, although their expression patterns varied. The occurrence of DQ2.5-Glia-α1a and DQ2.5-Glia-α3 was higher than that of DQ2.5-Glia-α2 and DQ2.5-Glia-α1b across all genotypes. Since a higher quantity of toxic alpha-gliadin epitopes is associated with increased immunogenicity in individuals susceptible to celiac disease, we measured the frequency of the most toxic alpha-gliadin epitopes among different synthetic lines to estimate the overall immunogenic load of our lines. Generally, the immunogenic load of lines with the D-genome originating from *Ae. crassa* was much lower than those with the D-genome from *Ae. tauschii*. In this way, the *Ae. tauschii* derived lines 5 and 6 contained higher levels of toxic alpha-gliadin epitopes, while lines 3, 4, and 7 (derived from *Ae. crassa*) contained the lowest levels of toxic peptides. We conclude that replacing the bread wheat D-genome with that of the *Ae. crassa* may help lower the gluten immunogenicity in the deriving synthetic wheat lines.

## 1 Introduction

Bread wheat (*Triticum aestivum* L., 2*n* = 6*x* = 42) is an important staple crop for the human diet and livestock feed with an annual production of 760 million tons in the world showing 30% of all harvested cereals (FAO, 2022). Although wheat contains a relatively low amount of protein (8–15%), it still remains the most significant source of protein in the human diet mainly due to its adaptability to various agricultural environments, convenience in storage, nutritional benefits, and the ability of its flour in processing of numerous tasty and satisfying foods (Shewry, 2009; Shewry and Hey, 2015). It has been widely shown that the functional characteristics of dough, which is essential for making various types of end products, are largely linked to gluten, which accounts for 80% of total wheat storage proteins. The major components of gluten are gliadins and glutenins, both of which accumulate in the endosperm during the graining phase (Shewry et al., 2002). Gluten proteins, also known as prolamins, are the water-insoluble component of wheat seed-storage proteins. They are rich in proline and glutamine and are made up of high- and low- molecular weight polymeric glutenin subunits (HMW-GSs and LMW-GSs) as well as monomeric gliadins (α/β-, γ-, and ω-gliadins), both of which contribute to the breadmaking quality of wheat flour (Shewry, 2009, Shewry, 2019). Despite the significant importance of gluten proteins in breadmaking properties, certain epitopes have been isolated from wheat gluten which can survive proteolysis in the human gastrointestinal tract and stimulate the immune response in genetically susceptible patients with celiac disease (CD). CD is a T cell mediated chronic inflammatory condition and an autoimmune enteropathy of the small intestine triggered by specific gluten peptides that are deamidated by tissue transglutaminase 2 (tTG2) in the human digestive system (Sollid, 2002; Tye-Din et al., 2010; Sollid et al., 2012; Ludvigsson et al., 2013). CD has an average global prevalence of 0.7% to 3% (Catassi et al., 2014; Singh et al., 2018). Currently, the only way to prevent CD symptoms is to remove wheat from the diet and consume gluten-free products. This is quite challenging since gluten is present in many food products due to its viscoelastic and binding properties (El Khoury et al., 2018; Jouanin et al., 2018; Shewry, 2019).

These immunogenic peptides contain highly specific cores that are at least nine amino acids long (Shan et al., 2002) and are presented to CD4 T cells by the human leukocyte antigen HLA-DQ2 and DQ8 haplotypes. This process initiates an immune response in the small intestine, resulting in a series of symptoms such as intestinal inflammation, villus atrophy, and nutrient malabsorption (Sollid, 2002; Ludvigsson et al., 2013). Among HLA-DQ molecules, HLA-DQ2.5 creates more stable complexes with gluten T-cell epitopes and is connected with a higher level of risk (Fallang et al., 2009). To date, several HLA-DQ2.5 epitopes have been identified in gluten proteins, primarily located in the gliadin fraction of gluten (Sollid et al., 2020).

Immunogenic epitopes are specifically found in α-, γ-, and ω-gliadins, as well as to a lesser degree in LMW-GSs. Alpha-gliadins are encoded by a multigene family located at the Gli-2 loci on the short arms of homoeologous chromosomes 6. Indeed, the tandem arrangement of a large number of alpha-gliadin duplicated genes at the Gli-2 loci with a 25 to even 150 copies per haploid genome, adds to the complexity of this gene family, making it challenging to obtain high-quality sequences and interpret related genes (Harberd et al., 1985; Huo et al., 2018). It has been estimated that up to 87 of alpha-gliadins in wheat terminates with internal stop codons, and are classified as pseudogenes (Anderson and Greene, 1997; van Herpen et al., 2006). In wheat, alpha gliadins are an important component of gluten, comprising an estimated 15–30% of total seed storage proteins. Alpha gliadins contain three major CD immunogenic peptides: the p31-43 peptide, which is associated with the immune response in CD non-T cell-dependent and initiates the development of CD (Maiuri et al., 2003); the 33-mer peptide, which shows higher immunogenicity and contains six overlapping copies of two toxic alpha-gliadin epitopes DQ2.5-Glia-α1 (PFPQPELPY), and DQ2.5-Glia-α2 (PQPELPYPQ); and the DQ2.5-Glia-α3 (FRPEQPYPQ) peptide, which is an epitope with lower immunogenicity and partially overlaps with the 33-mer toxic region (Anderson et al., 2000; Arentz-Hansen et al., 2000; Sollid et al., 2020; Marín-Sanz et al., 2023). Among the major epitopes, DQ2.5-Glia-α1 is the one that causes inflammation in the majority of CD patients (Qiao et al., 2004). These canonical toxic peptides and their variations among the alpha-gliadin genes are genome specific and largely located at the Gli-2 locus of the D-genome in hexaploid wheat (Salentijn et al., 2009; Jouanin et al., 2019). Moreover, it is believed that the D subgenome originated from a subgroup of *Ae. tauschii* during the long evolutionary history of common wheat (Halstead-Nussloch et al., 2021; Schaart et al., 2021).

Reducing the amount of CD epitopes in wheat products may lower the risk of immune system sensitization in those who are genetically predisposed to CD. There is significant variation in CD immunogenicity among different wheat varieties that is closely associated with the differences in CD-immunogenic epitopes found in the gluten proteins (Spaenij–Dekking et al., 2005). Recruiting D subgenomes from different Aegilops species may also be promising for producing amphiploids with lower CD immunogenicity. Thus, precise evaluation of the quantity and quality of CD epitopes in gluten is a key factor for breeding and selecting wheat varieties with significantly lower immunogenic potential for causing CD. Currently, sequencing of RNA amplicons related to the toxic region of alpha-gliadin genes has promisingly made it possible to measure the differences in celiac disease epitopes among different wheat varieties (Salentijn et al., 2013; Marín-Sanz et al., 2023). Commonly, the total RNA of developing seeds is extracted and the N-terminal region of immunogenic alpha-gliadins is sequenced using next generation sequencing (Salentijn et al., 2013).

The evolution of common wheat has been restricted to a few natural crossing events between *T. turgidum* and *Ae. tauschii* (the D-genome donor) in a closed geographical region. This evolutionary bottleneck along with the intensive selection during the breeding processes in the current decades has led to a narrow genetic diversity within the common wheat D-genome (Kumar et al., 2019). Thus, developing new synthetic wheat lines by crossing durum wheats with various D-containing Aegilops species may enhance wheat diversity and help develop wheats with improved gluten toxicity (Kishii, 2019; Kumar et al., 2019). Furthermore, studies have shown that in some Aegilops accessions, the alpha-gliadins do not contain the intact 33-mer epitope and could be utilized in developing synthetic hexaploid wheats with lower or no immunogenic gluten (Schaart et al., 2021).

In a previous work, we developed different synthetic wheats through hybridization between tetraploid wheats and Aegilops species containing D-genome from *Ae. tauschii*, *Ae. crassa*, and *Ae. ventricosa* (Mirzaghaderi et al., 2020). Here, we aimed to study the toxic region of alpha gliadins in eight synthetic wheat lines (**Table 1**) through sequencing of their RNA amplicons to compare the frequency and variation of CD epitopes among the related genotypes. This RNA amplicon expands all three major immunogenic peptides: p31-43, the 33-mer region, and DQ2.5-Glia-α3, offering an excellent approach for exploring the structural complexity of the most immunogenic alpha-gliadins region and identifying wheat lines with a reduced content of immunogenic gluten.

**Table 1:** Synthetic wheat lines, their cross combinations, and corresponding genomes/subgenomes. In the final column the number of intact 33-mer peptide is shown for each line.

Synthetic wheat lines	Pedigree	Genome	Intact 33-mer (No.)
Line 1	(*T. durum* ‘40’ × *Ae. tauschii* ‘299’) × *T. aestivum* ‘Pishgam’	AABBDD	0
Line 2	((*T. dicoccum* ‘Javanmard’ × *T. dicoccoides* ‘Seydsaleh’) × *Ae. tauschii* ‘AE 1211’) × *T. aestivum* ‘Pishgam’	AABBDD	607
Line 3	(*T. durum* ‘40’ × *Ae. crassa* ‘1873’)	AABBD^1^D^1^X^cr^X^cr^	187
Line 4	(*Ae. crassa* ‘S’ × *T. durum* ‘6268’)	AABBD^1^D^1^X^cr^X^cr^	0
Line 5	(*T. dicoccum* ‘TazeabadAliabad’ × *Ae. tauschii* ‘299’)	AABBDD	28
Line 6	(*T.dicoccoides* ‘IG127697’ × *Ae. tauschii* ‘299’)	AABBDD	0
Line 7	(*Ae. crassa* ‘B’ × *T. durum* ‘6268’)	AABBD^1^D^1^X^cr^X^cr^	0
Line 8	(*Ae. ventricosa* ‘1522’ × *T. durum* ‘13’)	AABBD^v^D^v^N^v^N^v^	0

## 2 Materials and methods

### 2.1 Plant material

A total of eight synthetic wheats, produced from different cross combinations between tetraploid wheats and Aegilops species containing D-genome [**Table 1**; (Mirzaghaderi et al., 2020)] were chosen. For each genotype, 12 seeds were planted in three pots containing loam soil (four seeds per pot). To ensure vernalization and more even flowering, the seedlings were first placed in a growth chamber at 4°C for 1 month, and then kept in a greenhouse with photoperiod of 16 hours light: 8 hours dark at 22°C. All pots were watered once a week and kept under the same conditions until the initiation of flowering and sampling.

### 2.2 RNA extraction and cDNA synthesis

Seeds were sampled during grain filling at 21 days post-anthesis when the kernels were at milk to soft dough stage. Seeds were immediately frozen in liquid nitrogen, and stored at -80°C. RNA isolation was performed using the single step extraction method by acid guanidinium thiocyanate–phenol–chloroform developed by (Chomczynski and Sacchi, 2006). Briefly, RNA was extracted from at least five immature seeds of each single plant by adding 1 ml of denaturing solution (4 M guanidinium thiocyanate, 25 mM sodium citrate, pH 7.0, 0.5% (wt/vol) *N-*laurosylsarcosine (Sarkosyl) and 0.1 M 2-mercaptoethanol) into the crushed grains in liquid nitrogen, and followed by phase separation in an acidic pH to ensure the separation of RNA from DNA and proteins. After precipitation of extracted RNA in isopropanol, RNA was washed using 75% ethanol. The RNA pellet was air-dried and then dissolved in 50 µl of DEPC-treated water and stored at -80°C until cDNA synthesis. RNA extraction using the protocol mentioned above at acidic pH usually results in pure RNA. To test whether our extracted RNA samples are free of DNA, we performed an additional PCR on each RNA sample using alpha-gliadin primers. No band was detected after running the PCR products on agarose gel (**Supplementary Figure S1A**), so no DNase treatment was applied. The quality and quantity of extracted RNA were measured using agarose gel electrophoresis and NanoDrop spectrophotometry. For all samples, the concentration of RNA was equalized. Three biological RNA samples were then pooled for each genotype, and 1 µg of the mixed RNA was used for cDNA synthesis. cDNA was prepared using the TaKaRa kit (Otsu, Shiga, Japan) according to the vendor’s protocol.

### 2.3 Amplification of the alpha-gliadin amplicon

**Figure 1:**
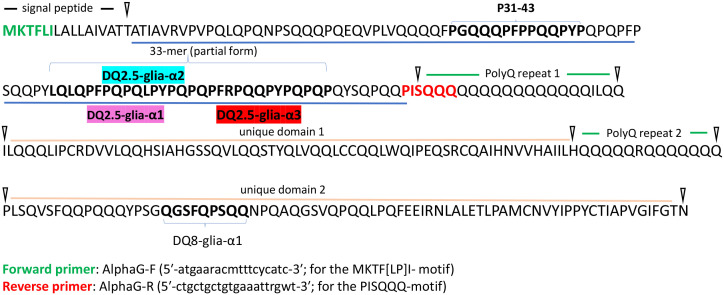
Amino acid sequence of a typical alpha-gliadin (ADD17012.1). The position of three major CD epitopes DQ2.5-Glia-α1, -α2 and -α3 are highlighted on the sequence. The location of the used forward and reverse PCR primers is indicated on the sequence with green and red colors. The first repetitive domain harboring canonical CD epitopes of alpha-gliadins is underlined in blue. This figure is modified from (Salentijn et al., 2013).

Alpha-gliadin sequences were amplified from cDNA samples in two steps. The first amplification was performed using gene specific primers of alpha-gliadin; AlphaF (5′-atgaaracmtttcycatc-3′; for the MKTF[LP]I- motif) and AlphaR (5′- ctgctgctgtgaaattrgwt-3′; for the PISQQQ-motif) (**Figure 1**; **Supplementary Figure S1C**). Both forward and reverse primers were chosen from (Salentijn et al., 2013), which specifically amplify the repetitive domain of a typical alpha gliadin gene coding the major CD epitopes including the p31-p43 peptide and the toxic 33-mer fragment containing DQ2.5-Glia-α1, DQ2.5-Glia-α2, and DQ2.5-Glia-α3 peptides (**Figure 1**, underlined in blue). To lower the amplification bias, amplification of alpha-gliadin amplicon was performed on two different PCR thermocyclers. PCR reaction was prepared in 10 µl including 25 ng template cDNA, 5 pmol of each primer, 2.5 mM of each dNTP, 2.5 mM MgCl_2_ and 0.5 U Taq polymerase. The PCR cycling parameters included an initial step at 94°C for 5 min, followed by 30 cycles of (30 seconds at 94°C, 15 seconds at 50°C, and 30 seconds at 72°C), and a final extension at 72°C for 5 min. Then, for each sample, the PCR products (two technical replicates) were mixed thoroughly and diluted 1 to 10 using nuclease-free water. 1 µl of diluted PCR product from the first round of PCR was used as a template for the second round of amplification. The second round of PCR was performed in 10 µl reactions using fusion primers. Fusion primers included the sequences needed for Illumina sequencing, and a gene specific part. The forward fusion primer also contained an 8 bp ID sequence (barcode) specific for each genotype (**Supplementary Figure S1C**) to enable multiplexing. The second-round PCR condition was similar to the first PCR amplification. The PCR products from eight different genotypes were pooled, and 80 µl of the pooled PCR product was sent for paired-end Illumina amplicon sequencing (Novogene (UK) Company). The resulting reads belonging to each genotype could later be identified via the ID oligo sequence of the forward primer.

### 2.4 Analysis of alpha-gliadin amplicons and the expression profiles of CD epitopes

**Figure 2:**
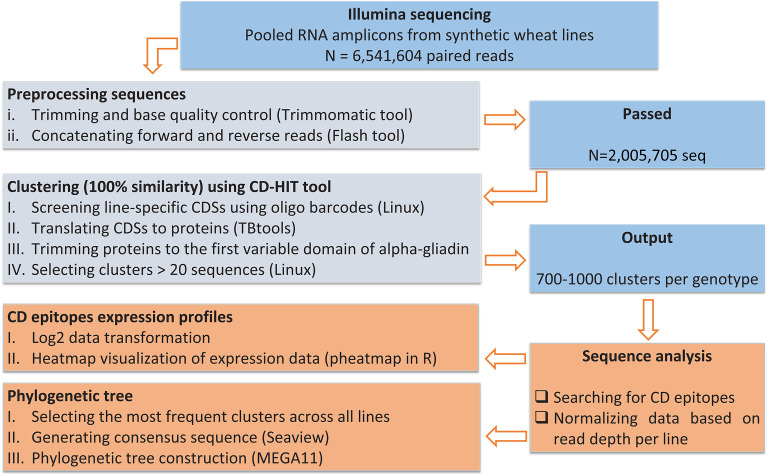
An overall pipeline for pre-processing and analyzing of alpha-gliadin amplicon sequencing data.

**Figure 3:**
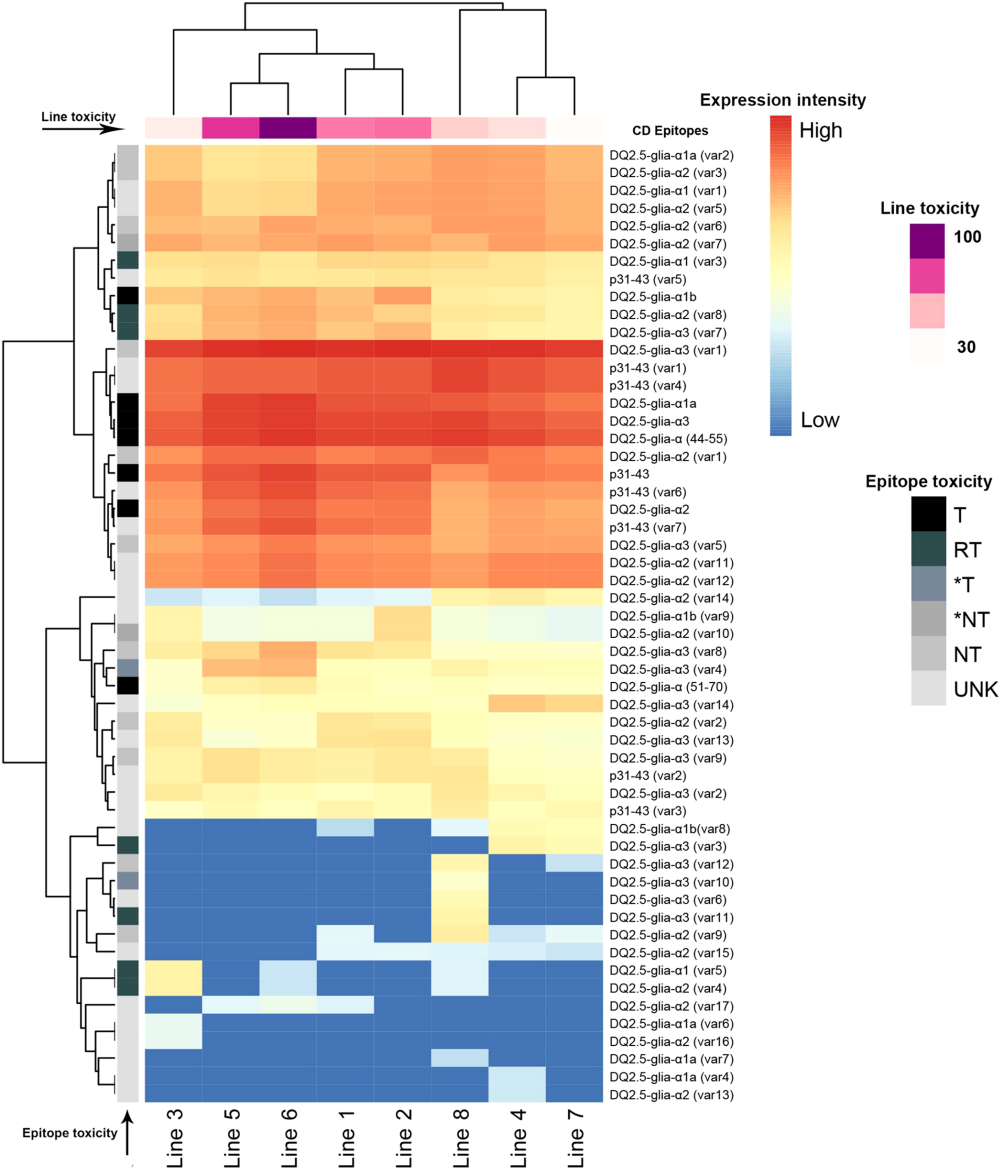
Expression patterns of CD epitopes in the studied synthetic wheat lines. The epitope toxicity is shown in grays (T: toxic peptides, RT: decreased toxicity, *T, highly likely toxic, *NT, highly likely not toxic, NT, non-toxic and UNK, unknown). Lines were clustered based on the cumulative expression of the toxic (T), decreased toxicity (RT), and highly likely toxic (*T) epitopes from dark purple (highly toxic) to light pink (less toxic). Lines pedigree is indicated in **Table 1**.

An overall pipeline for pre-processing and analyzing of alpha-gliadin amplicon reads is shown in **Figure 2**. The paired-end Illumina sequencing of the pooled alpha-gliadin amplicons resulted in a total of 6,541,604 paired-end reads (250 bp length per read). The alpha-gliadin reads were first checked for their quality using FastQC (Andrews, 2010). Then, trimming and base quality control of the reads were performed using trimmomatic tool (Bolger et al., 2014). The forward and reverse reads were concatenated/assembled using FLASH tool (Magoč and Salzberg, 2011) in Linux. The concatenated sequences belonging to each genotype were separated using the genotype-specific oligonucleotide barcodes embedded in the forward primers (**Supplementary Figure S1C**). Next, for each genotype, the sequences were further trimmed to remove the genotype identifier barcode, signal peptide, and the forward and reverse primers (**Figure 1**). These preprocessed codding sequences (CDSs) were translated to proteins using TBtools (Chen et al., 2023). Protein sequences were then clustered using CD-HIT at 100% identity (Li and Godzik, 2006) in Linux, and the representative sequences with more than 20 members were chosen for alpha-gliadin immunogenic peptides analysis. The frequency of CD epitopes was calculated based on the abundance of the amplicons containing them. Briefly, different alpha-gliadin epitopes, including the canonical epitopes and variants with one or two mismatches were searched only in the representative clusters with more than 20 members. For each single representative protein, the number of identified epitopes was multiplied by the number of its cluster members to determine the abundance of epitopes across the clusters. To estimate the abundance of a specific epitope across the lines, the frequency of that epitope was summed for all clusters. Subsequently, the number of each calculated epitope was normalized based on read depth per line, and represented as relative frequency/expression rate and their log 2 values (**Supplementary Table 1**) were visualized by a heatmap in R software using the pheatmap package (Kolde, 2019). We also coded the alpha-gliadin epitopes based on their previously reported toxicity information into three groups (T: toxic, RT: decreased toxicity, *T: highly likely toxic) and assigned scores (3 for T, 2 for RT, and 1 for highly likely toxic) to estimate the overall toxicity load of each line (**Figure 3**; **Supplementary Table 2**). To achieve this, the number of peptides for each group was multiplied by its corresponding score. Then, the line with the highest toxicity score was assumed 100% toxic, and the toxicity percentages of other lines were calculated relative to this line (**Supplementary Table 2**).

### 2.5 Phylogenetic tree of synthetic wheat lines

To construct the phylogenetic tree, first, the ten most prevalent clusters of alpha-gliadin proteins were chosen in each genotype (**Supplementary Table 3**). Then, the consensus protein sequences were created for each line separately using SeaView tool version 4 (Gouy et al., 2010). Logos for the alignment of the consensus proteins were generated using online WebLogo application (Crooks et al., 2004). A phylogenetic tree was also constructed using the consensus sequences in MEGA11 software (Tamura et al., 2021) with neighbor-joining method and bootstrapping of 1000 replicates.

### 2.6 Acid-PAGE analysis of gliadins

To analyze the gliadin banding patterns of synthetic lines, we performed Acid-PAGE. First, gliadin proteins were extracted using the method described by Khan et al. (1985) with slight modifications. 200 µl of 70% ethanol was added to 0.1 g of fresh milled flour in a 1.5 ml tube. The samples were then vortexed every 10 min for 1 hour at room temperature and centrifuged at 13,000 rpm for 15 minutes. For each genotype, 20 µl of supernatant was transferred to a new 1.5 ml tube and heated at 70°C in a water-bath to form a protein pellet. Then, 20 µl of 5% acetic acid and 20 µl of sample solution (4.5 M urea solution prepared in 5% acetic acid) were added to the pellets and vortexed for 10 seconds. The prepared protein samples were stored at -20°C until use. Acid-PAGE was performed according to Watry et al. (2020). Proteins were heated at 70°C for 10 minutes before running the gel. In each well, 6 µl of proteins were loaded onto a 10% acrylamide gel and run for 90 min at 400 V. The gel image was captured using a smartphone camera of 108 MP (Xiaomi Poco X4).

## 3 Results

### 3.1 Alpha gliadin RNA sequencing and sequence analysis

Eight different synthetic wheat lines resulted from different cross combinations (**Table 1**), were examined for their alpha-gliadin protein composition and CD epitope content. For this, RNA was extracted from immature seeds and converted into cDNA. The first variable repetitive domain of alpha-gliadins (**Figure 1**, underlined in blue) harboring the three major CD epitopes DQ2.5-glia-α1, DQ2.5-glia-α2 and DQ2.5-glia-α3 (**Figure 1**, highlighted in magenta, cyan and red, respectively), was amplified from each genotype, and the pooled PCR products were sequenced using Illumina platform. Because we used a specific barcode sequence in the used primers for amplification of each genotype (**Supplementary Figure S1C**), it was possible to pool the PCR products before amplicon sequencing. In this way, the sequences specific to each line could later be identified and separated during data processing. A summary of data pre-processing and analyzing the alpha-gliadin epitope composition across the lines is presented in **Figure 2**.

Overall, 6,541,604 paired reads (an average of 600,000-800,000 reads per plant, 250 bp in size) were generated from the alpha-gliadin amplicons across all samples. After trimming, base quality control and concatenation/assembling of forward and reveres reads, collectively 2,005,705 CDSs (an average of 200,000-300,000 sequences per plant, 192-432 bp in size) were generated, from which 111,336 sequences contained stop codon(s) and were discarded from the analysis. The remaining sequences (1,894,369 CDSs) were used for analyzing and comparing the alpha gliadin CD epitopes among the genotypes. CDSs belonging to each line were separated using the specific barcode sequence (**Supplementary Figure S1C**). The sequences were then translated to proteins in TBtools, and the protein sequences of each line were clustered using CD-HIT tool with a threshold of 100% similarity. As a result, a total of 744,981 clusters were produced across all plants, which were later minimized by selecting only those clusters with at least 20 members. In this way, 700-1000 clusters were remained for each plant (**Supplementary Table 4**).

### 3.2 Alpha-gliadin epitopes expression profiles

For analyzing the expression pattern of alpha-gliadin epitopes among different synthetic wheat lines, first, a list of all previously predicted/identified alpha-gliadin epitopes, the canonical forms, all those variants with one or two mismatches and their toxicity information (if available) were retrieved from the literature (Shan et al., 2002; Ozuna et al., 2015; Wang et al., 2017; Altenbach et al., 2020; Sollid et al., 2020; Schaart et al., 2021; Marín-Sanz et al., 2023). These epitopes (121 in total) were searched in the alpha-gliadin protein clusters in each line. Overall, 54 epitopes were found in the analyzed lines (**Table 2**): Eleven epitopes were DQ2.5-Glia-α1 and its variants. Eighteen were DQ2.5-Glia-α2 and its variants, among which variants 15, 16 and 17 containing only one mismatch, were reported for the first time. Sixteen were DQ2.5-Glia-α3 and its variants, of which, a new variant with one mismatch (variant 14) was identified. Hence, four new variants were identified which have been highlighted in **Table 2**. Regarding the P31-43 peptide, beside the canonical epitope, six variants were detected. All the canonical epitopes (bolded in **Table 2**) were present across all the synthetic lines, though with different frequencies. However, the distribution and frequency of the variants varied significantly among the lines (**Table 2**).

**Table 2:** Canonical alpha-gliadin epitopes, related variants and their toxic nature in each synthetic line.

Epitopes	Toxicity	Peptides	Synthetic Wheat Lines							
			L1	L2	L3	L4	L5	L6	L7	L8
**DQ2.5-glia-α1a**	T	**PFPQPQLPY**	1	1	1	1	1	1	1	1
**DQ2.5-glia-α1b**	T	**PYPQPQLPY**	1	1	1	1	1	1	1	1
DQ2.5-glia-α1 (var1)	UNK	PFLQPQLPY	1	1	1	1	1	1	1	1
DQ2.5-glia-α1a (var2)	NT	PFPQPQLSY	1	1	1	1	1	1	1	1
DQ2.5-glia-α1 (var3)	RT	PFSQPQLPY	1	1	1	1	1	1	1	1
DQ2.5-glia-α1a (var4)	UNK	PFPQPQLPH	0	0	0	1	0	0	0	0
DQ2.5-glia-α1 (var5)	RT	PFPQLQLPY	0	0	1	0	0	1	0	1
DQ2.5-glia-α1a (var6)	UNK	PFPQPPLPY	0	0	1	0	0	0	0	0
DQ2.5-glia-α1a (var7)	UNK	PFPQLQQPY	0	0	0	0	0	0	0	1
DQ2.5-glia-α1b(var8)	UNK	PYPQPQLFP	1	0	0	1	0	0	1	1
DQ2.5-glia-α1b (var9)	UNK	PYPQPHLPY	1	1	1	1	1	1	1	1
**DQ2.5-glia-α2**	T	**PQPQLPYPQ**	1	1	1	1	1	1	1	1
DQ2.5-glia-α2 (var1)	NT	PQPQLPYSQ	1	1	1	1	1	1	1	1
DQ2.5-glia-α2 (var2)	NT	SQPQLPYSQ	1	1	1	1	1	1	1	1
DQ2.5-glia-α2 (var3)	NT	PQPQLSYSQ	1	1	1	1	1	1	1	1
DQ2.5-glia-α2 (var4)	RT	PQLQLPYPQ	0	0	1	0	0	1	0	1
DQ2.5-glia-α2 (var5)	UNK	LQPQLPYSQ	1	1	1	1	1	1	1	1
DQ2.5-glia-α2 (var6)	NT	FPPQLPYPQ	1	1	1	1	1	1	1	1
DQ2.5-glia-α2 (var7)	*NT	FLPQLPYPQ	1	1	1	1	1	1	1	1
DQ2.5-glia-α2 (var8)	RT	PQPQLPYLQ	1	1	1	1	1	1	1	1
DQ2.5-glia-α2 (var9)	NT	PQPQLPYSH	1	0	0	1	0	0	1	1
DQ2.5-glia-α2 (var10)	*NT	PQPHLPYPQ	1	1	1	1	1	1	1	1
DQ2.5-glia-α2 (var11)	UNK	PQPQPQYPQ	1	1	1	1	1	1	1	1
DQ2.5-glia-α2 (var12)	UNK	PQPQYPQPQ	1	1	1	1	1	1	1	1
DQ2.5-glia-α2 (var13)	UNK	PQPQLPHSQ	0	0	0	1	0	0	0	0
DQ2.5-glia-α2 (var14)	UNK	SQPQLPYLQ	1	1	1	1	1	1	1	1
DQ2.5-glia-α2 (var15)	UNK	LQPQLPYPQ	1	1	0	1	0	0	1	1
DQ2.5-glia-α2 (var16)	UNK	PQPPLPYPQ	0	0	1	0	0	0	0	0
DQ2.5-glia-α2 (var17)	UNK	PQPQLPYHQ	1	0	0	0	1	1	0	0
**DQ2.5-glia-α3**	T	**FRPQQPYPQ**	1	1	1	1	1	1	1	1
DQ2.5-glia-α3 (var1)	NT	FPPQQPYPQ	1	1	1	1	1	1	1	1
DQ2.5-glia-α3 (var2)	UNK	FSPQQPYPQ	1	1	1	1	1	1	1	1
DQ2.5-glia-α3 (var3)	RT	FLPQQPYPQ	0	0	0	1	0	0	1	0
DQ2.5-glia-α3 (var4)	*T	FPQQQPYPQ	1	1	1	1	1	1	1	1
DQ2.5-glia-α3 (var5)	NT	FPPQQSYPQ	1	1	1	1	1	1	1	1
DQ2.5-glia-α3 (var6)	UNK	FQPQQPYPQ	0	0	0	0	0	0	0	1
DQ2.5-glia-α3 (var7)	RT	FRPQQSYPQ	1	1	1	1	1	1	1	1
DQ2.5-glia-α3 (var8)	NT	FPPQQPYPH	1	1	1	1	1	1	1	1
DQ2.5-glia-α3 (var9)	NT	FPPQQPYLQ	1	1	1	1	1	1	1	1
DQ2.5-glia-α3 (var10)	*T	FRPQKPYPQ	0	0	0	0	0	0	0	1
DQ2.5-glia-α3 (var11)	RT	FRQQQPYPQ	0	0	0	0	0	0	0	1
DQ2.5-glia-α3 (var12)	NT	FPPQQPYTQ	0	0	0	0	0	0	1	1
DQ2.5-glia-α3 (var13)	UNK	FRPQQLYPQ	1	1	1	1	1	1	1	1
DQ2.5-glia-α3 (var14)	UNK	FRPQQPFPQ	1	1	1	1	1	1	1	1
**p31-41**	T	PGQQQPFPPQQPY	1	1	1	1	1	1	1	1
P31-43(var1)	UNK	LGQQQPFPPQQPY	1	1	1	1	1	1	1	1
P31-43(var2)	UNK	LGQQQQFPPQQPY	1	1	1	1	1	1	1	1
P31-43(var3)	UNK	LGQQQPFRPQQPY	1	1	1	1	1	1	1	1
P31-43(var4)	UNK	LGQ*QPFPPQQPY	1	1	1	1	1	1	1	1
P31-43(var5)	UNK	LGQQQSFPPQQPY	1	1	1	1	1	1	1	1
P31-43(var6)	T	PGQQQPFPPQQPYPQPQPF	1	1	1	1	1	1	1	1
P31-43(var7)	T	PGQQQPFPPQQPYPQPQPFPSQQPY	1	1	1	1	1	1	1	1
DQ2.5-glia-α (44-55)	T	PQPQPFPSQQPY	1	1	1	1	1	1	1	1
DQ2.5-glia-α (51-70)	T	SQQPYLQLQPFPQPQLPY	1	1	1	1	1	1	1	1
**Total epitopes**			42	39	42	44	39	41	43	48

Numbers 1 and 0 in the last column indicate whether the represented epitopes are found in the given line or not, respectively. The total number of CD epitopes is also shown for different synthetic genotypes at the final line of the table. The canonical epitopes and their peptide sequences are written in bold font. The epitopes of the new alpha-gliadin variants are highlighted in gray. Variants with one or two mismatches their residues are written in red. The green color in the second residue of the DQ2.5-Glia-α1b peptide shows that the related peptide is a canonical variant of DQ2.5-Glia-α1. L and the given number indicating different synthetic wheat line. The toxicity of different epitopes is shown with different symbols; T stands for (toxic peptides), RT (decreased toxicity), *T (highly likely to be toxic), *NT (highly likely to be not toxic), NT (non-toxic) and UNK (unknown).

Specifically, the major and most immunogenic CD epitopes DQ2.5-Glia-α1a (PFPQPQLPY), DQ2.5-Glia-α1b (PYPQPQLPY), DQ2.5-Glia-α2 (PQPQLPYPQ), and DQ2.5-Glia-α3 (FRPQQPYPQ) which are located in the 33-mer region of *Ae. tauschii* alpha-gliadin (GenBank: ADD17012.1) (**Figure 1**), were expressed across all synthetic wheat lines, albeit with different frequencies (**Figure 3**, **Table 2**; **Supplementary Table 1**). Collectively, the expression profile of DQ2.5-Glia-α1a and DQ2.5-Glia-α3 were higher than that of DQ2.5-Glia-α2 and DQ2.5-Glia-α1b. Among these canonical epitopes, the expression pattern of DQ2.5-Glia-α1b was the lowest especially in Lines 4, 7 and 8. The expression of each alpha-gliadin epitopes also differed across different synthetic lines. DQ2.5-Glia-α1 had the highest expression frequency in line 5 and 6, while the lowest frequency was observed in *Ae. crassa* derived lines 3, 4, and 7. DQ2.5-Glia-α2 was expressed with higher intensity in line 6 and with lower intensity in line 7 and 8. DQ2.5-Glia-α3 differed from other alpha-gliadins in its expression pattern among the different lines. All lines significantly expressed DQ2.5-Glia-α3 epitope with high intensity, except for lines 3 and 7 (**Figure 3**; **Supplementary Table 1**). Our data also showed that a variant of alpha-gliadin epitope, DQ2.5-Glia-α3 (var1, FPPQQPYPQ) showed the highest expression rate across all lines. The newly identified variants of DQ2.5-Glia-α2 (var15, var16, and var17) were rarely expressed, and were only found in some synthetic lines. In contrast, the new variant of DQ2.5-Glia-α2 (var14) was found in all lines, showing higher expression levels in lines 4 and 7 (**Figure 3**; **Supplementary Table 1**). The p31–43 peptide, which induces the innate immune response in patients susceptible to CD, and its variants (var1 and var4) had higher expression compared to other variants. It had a higher frequency in line 8 and a lower frequency in line 3. In addition to analyzing each of these CD epitopes separately, we also searched for the intact 33-mer region across the synthetic lines. The intact form of the 33-mer region (LQLQPFPQPQLPYPQPQLPYPQPQLPYPQPQPF; AFX69628.1) was found only in three lines (Lines 2, 3, and 5), showing the highest expression rate in line 2 and a lower expression rate in line 5 (**Table 1**; **Supplementary Table 5**).

The wheat lines were also compared for the frequency of the toxic alpha-gliadin epitopes and those with the lower and higher toxicity were identified. For this, the normalized frequency of toxic (T), decreased toxicity (RT) and highly likely to be toxic (*T) epitopes were multiplied by 3, 2 and 1 scores relative to their toxic intensity, respectively. Then, the toxicity of the lines was expressed as a percentage relative to the line with the highest toxicity score as discussed in the methods section (**Figure 3**; **Supplementary Table 2**). This generally showed that the major and toxic alpha-gliadin peptides were present in all lines, however, different synthetic lines exhibited dramatic variability in their toxicity (**Supplementary Table 2**). Based on the frequency of toxic alpha-gliadins across the lines and our assumption (considering the line with the highest frequency of toxic CD epitopes as 100% toxic), the highest toxicity percentages were found for line 6 (100% with 435,534 T, RT, and *T peptides), followed by line 5 (~80% with 350,618 T, RT, and *T peptides). These two lines have received the D-genome from *Ae. tauschii* ‘299’, but their maternal parents belong to different emmer wheats (**Figure 3**, **Table 1**). Lines 1 and 2 were not significantly different for their CD toxicity (67% vs 69%, respectively). Both lines have different maternal parents, but they have been crossed with *Ae. tauschii*, and experienced the same backcross event with the hexaploid wheat *T. aestivum* ‘Pishgam’. Line 8 with the D-genome from *Ae. ventricosa* possessed a toxicity percentage of 49% and was the fourth line regarding CD toxicity. Lines 3, line 4 and 7, with the toxicity percentages of 37%, 42% and 32%, respectively, had the lowest contents of toxic peptides (**Supplementary Table 2**). Interestingly, these three lines received their D genome from *Ae. crassa* and their AB subgenomes from the same tetraploid genotype of *T. durum*, except for line 3 (**Figure 3**, **Table 1**).

We also found that the frequency of epitopes with reduced toxicity varied among different synthetic lines, and some epitopes were not detected in certain lines (**Table 2**; **Supplementary Table 1**). Epitopes DQ2.5-Glia-α1 (var3) PFSQPQLPY, DQ2.5-Glia-α2 (var8) PQPQLPYLQ, and DQ2.5-Glia-α3 (var7) FRPQQSYPQ were found in all lines. Peptide PFSQPQLPY with the higher frequencies (each containing 2,850 and 2,785 peptides, correspondingly) was found in the synthetic lines 2 and 1. While the later peptides, PQPQLPYLQ and FRPQQSYPQ had a higher proportion in the synthetic line 6. Peptide FRQQQPYPQ was a variant of DQ2.5-Glia-α3 (var11) and was only present in line 8. Other peptides such as FRPQKPYPQ, FPPQQPYTQ, PFPQLQQPY and FQPQQPYPQ), which were mainly variants of DQ2.5-Glia-alpha3, expressed only in synthetic line 8 (**Table 2**; **Supplementary Table 1**).

### 3.3 Phylogenetic relationships of synthetic wheat lines

**Figure 4:**
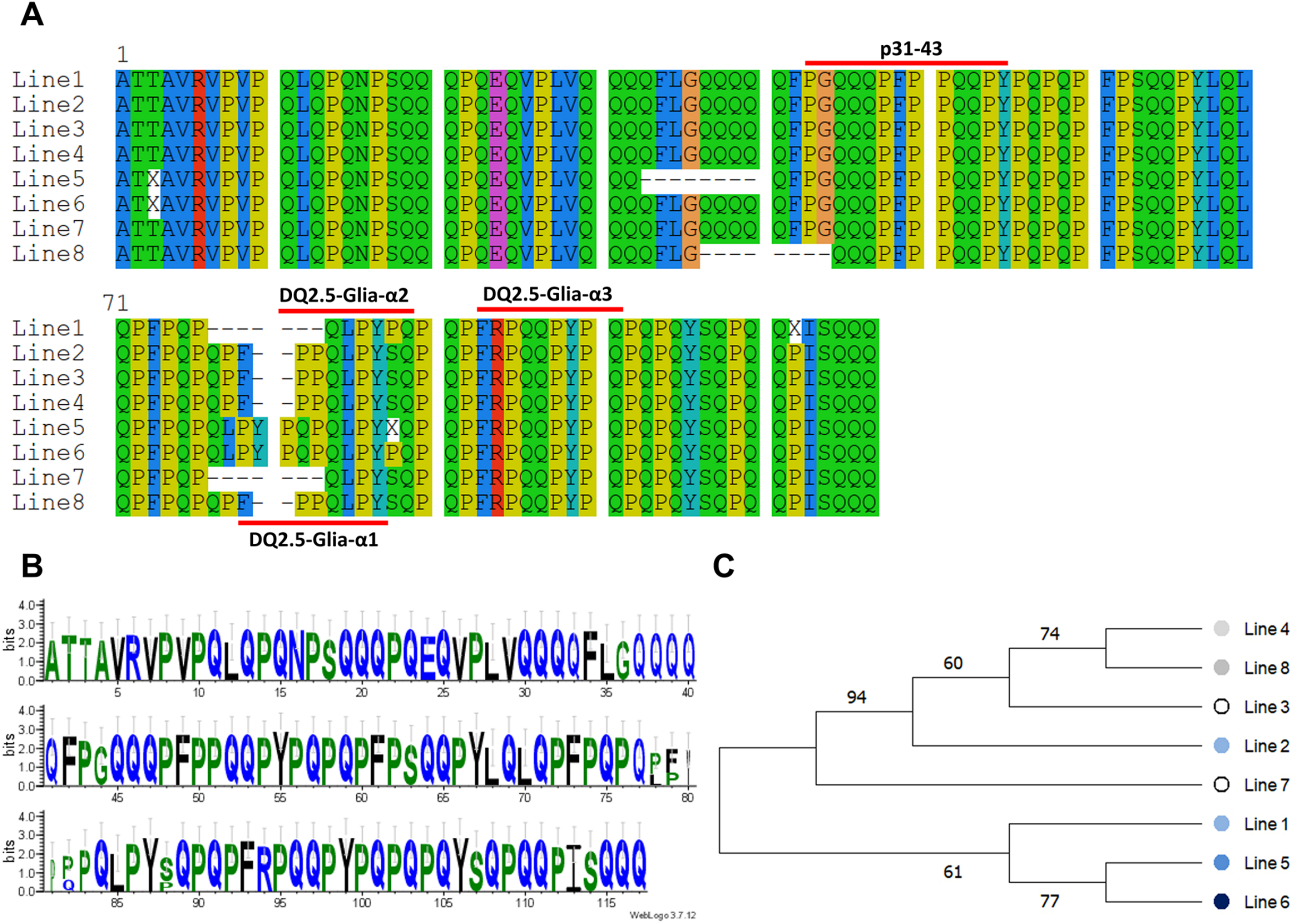
Analysis of alpha-gliadin consensus sequences in synthetic wheat lines. **(A)** Alignment pattern of the consensus sequences of the most common alpha-gliadin proteins in different synthetic lines. The alignment pattern of the consensus sequences shows that P31-43 peptide and DQ2.5-Glia-α3 are conserved across all synthetic lines. **(B)** A sequence logo of aligned the most abundant of alpha-gliadin proteins. **(C)** Phylogenetic relationships of different synthetic wheat lines based on the most frequent alpha-gliadin proteins in each line.

The phylogenetic relationship of the synthetic lines was constructed based on the consensus sequences of the most common clusters for each line. The alignment graph of these consensus protein sequences indicated that the peptides p31-43 (PGQQQPFPPQQPY) and one of the major CD epitopes DQ2.5-Glia-α3 (FRPQQPYPQ) were conserved across all genotypes (**Figure 4A**). Based on the logo of the consensus proteins, the amino acid residues were almost equally distributed among all synthetic lines (**Figure 4B**). The phylogenetic tree of the synthetic lines also rather matched the toxicity scores of the lines. In this way, the most toxic lines (line 5 and 6) as we previously identified based on their frequency of toxic alpha-gliadin epitopes (**Figure 3**, Line toxicity), both were grouped together, and had closer phylogenetic relationship (**Figure 4C**).

### 3.4 Acid-PAGE patterns of gliadins

We also assessed the gliadin proteins of our lines using Acid-PAGE and compared their banding patterns with those of Chinese spring wheat (*T. aestivum*). Though the banding patterns of ω-, β-, and γ-gliadins were not our concern in this study, they showed high variations across the genotypes (**Supplementary Figure S2**). The banding patterns of alpha-gliadins also differed among the lines and had one or two bands missing compared to Chinese spring wheat (**Supplementary Figure S2**, red arrows). Collectively, the lines with higher abundance of toxic CD epitopes (lines 5 and 6) and the line with relatively decreased toxicity (line 8) had only one missing band, while the lines with a lower toxicity (lines 3, 4 and 7) had two bands missing. These findings generally agreed with the results of sequencing data, indicating that lines with higher amounts of toxic CD epitopes had more alpha-gliadin bands compared to those with lower toxicity.

## 4 Discussion

The gluten component of wheat contains some immunogenic peptides that can stimulate a T cell response in individuals with celiac disease (CD), resulting in inflammation in the small intestine of about 2% of human population. Thus, developing new wheat varieties that are low in toxic gluten is a promising strategy to reduce the growing incidence of health issues related to the consumption of cereals and their products. Over the last few decades, there have been many efforts toward developing less immunogenic gluten wheat through RNAi and CRISPR/Cas technologies. Although these methods have successfully produced low-gluten wheats, the regulatory policies, especially within the European Union, poses a barrier to the short- or medium-term adoption of these varieties (Gil-Humanes et al., 2010, Gil-Humanes et al., 2014; Jouanin et al., 2019). Other efforts have focused on identifying less immunogenic varieties by screening the available bread wheats (Marín-Sanz et al., 2023). Due to the evolution of the wheat D-genome from very limited sources of Aegilops accessions, reaching varieties with free or low immunogenic gluten is a rare event. Thus, developing new synthetic wheat lines through different cross combinations of tetraploid wheats and unexplored polyploid Aegilops species containing the D-genome can circumvent these challenging policies and offer an alternative approach for producing fewer toxic wheats.

Various genomics and proteomics approaches including RNA and DNA amplicon sequencing, HPLC, SDS- and acid-PAGE have been employed to study the CD epitopes in the gliadin sequences of different wheats such as *Triticum turgidum*, *Triticum aestivum* and their ancestors by focusing on the most immunogenic region of alpha-gliadins. As well as, by using these methods, the varieties with low toxic gluten content have been successfully identified either from the available wheat varieties or from those which developed using gene editing and or gene transformation methods (Salentijn et al., 2013; Ozuna et al., 2015; Huo et al., 2018; Schaart et al., 2021; Marín-Sanz et al., 2023). In the present study, we aimed to evaluate and compare the content and composition of alpha-gliadins containing 33-mer peptides (which contains highly CD-toxic epitope variants in common wheats) through RNA amplicon sequencing among eight synthetic wheat lines developed in our previous research (Mirzaghaderi et al., 2020). All synthetic lines studied here, had all three major and the most immunogenic alpha-gliadin epitopes (DQ2.5-Glia-α1a (PFPQPQLPY)/DQ2.5-Glia-α1b (PYPQPQLPY); DQ2.5-Glia-α2 (PQPQLPYPQ); and DQ2.5-Glia-α3 (FRPQQPYPQ)) in the 33-mer region, indicating that the toxicity of gluten is still present in the developed lines. In our study, the occurrence of DQ2.5-Glia-α1a and DQ2.5-Glia-α3 was higher than that of DQ2.5-Glia-α2 and DQ2.5-Glia-α1b for all genotypes. Moreover, these epitopes were largely expressed in synthetic lines with D-genome from *Ae. tauschii* (Lines 5 and 6). Such a pattern has also been reported in *Ae. tauschii* accessions with a higher frequency than different bread wheat genotypes (Schaart et al., 2021; Marín-Sanz et al., 2023). Thus, we can conclude that the integration of these epitopes in our lines may, in part, come from the D-subgenome of *Ae. tauschii*. Regarding non-canonical variants, the FPPQQPYPQ peptide, a variant of the toxic DQ2.5-Glia-α3, contained an R to S substitution. Although this variant exhibited a higher expression frequency among all epitopes and synthetic lines, an earlier study showed that this type of substitution changes the toxic nature of the variant (Mitea et al., 2010). Therefore, the frequency of this variant was disregarded in evaluating the toxicity of our lines.

In the present research, the frequency of CD-inducing epitopes, whether the most toxic or those with reduced toxicity, varied significantly among different synthetic lines. There is a significant relationship between subgenome D and its potential for immunogenicity in common wheat (Halstead-Nussloch et al., 2021). It has also been shown that durum wheats and some Aegilops accessions, contrary to hexaploid wheats, do not contain the full 33-mer and are low in alpha-gliadins (Ozuna et al., 2015; Huo et al., 2018; Marín-Sanz et al., 2023). Our synthetic lines contained D-genomes from three different Aegilops species: *Ae. tauschii*, *Ae. crassa*, and *Ae. ventricosa*. Therefore, due to the integration of the D-genome from different sources (**Table 1**) in our synthetic lines, these variations in the number of CD epitopes are expected. For example, all synthetic lines with a D-genome from *Ae. crassa* and *Ae. ventricosa* had fewer toxic CD epitopes than those from *Ae. tauschii*. Although the lines derived from *Ae. crassa* generally contained lower amounts of toxic CD epitopes, it seems that they cause intraspecific variation in the resulting amphiploids (**Table 2**). This emphasizes the *Ae. crassa* potential in developing wheat with safer gluten content.

We also showed that the frequency of the P31-43 peptide was higher and more conserved across all synthetic lines than that of some canonical alpha-gliadin epitopes (**Figures 3**, **4**). It also did not have strong correlation with other epitopes among synthetic lines. Earlier studies have indicated that this peptide is less toxic compared to other canonical epitopes (Tye-Din et al., 2010; Vriz et al., 2021) and is primarily present in the A-subgenome, thus it occurs with high abundance in most wheat genotypes compared to some of the alpha-gliadin DQ2.5 epitopes (Maiuri et al., 2003; Halstead-Nussloch et al., 2021; Marín-Sanz et al., 2023).

We were also interested in determining whether the synthetic lines contain an intact form of the 33-mer peptide with six overlapping CD epitopes, as this form is a unique feature of the D genome alpha-gliadin in bread wheat. Interestingly, this form was found in some lines with D-genome from *Ae. tauschii* (Lines 2 and 5) and *Ae. crassa* (Line 3) at a low frequency. However, other lines (1, 4, 6, and 7), and the line with the D-subgenome from *Ae. ventricosa*, lacked the intact 33-mer region. Although this form of intact 33-mer has not been found in *Ae. tauschii* accessions in the previous research (Ozuna et al., 2015; Huo et al., 2018), newer studies have shown the presence of the 33-mer in some *Ae. tauschii* accessions (Schaart et al., 2021). In our study, not all lines with the D-subgenome from *Ae. tauschii* and *Ae. crassa* showed the presence of the intact form of the 33-mer peptide, indicating the potential of some Aegilops accessions for developing 33-mer-free gluten.

## 5 Conclusion

Here a number of synthetic amphiploids/wheats with diverse D genome were evaluated for their CD epitopes content and composition. Overall, the synthetic lines with the D-subgenome from *Ae. crassa* contained significantly lower levels of toxic alpha-gliadin CD-epitopes comparing to those with the D-subgenome from other Aegilops species. Therefore, this species may potentially be served as a valuable foundation in breeding programs aimed at developing hypoimmunogenic or possibly CD-safe bread wheat lines.

## Data Availability

The datasets supporting the conclusions of this article are available in the NCBI Short Read Archive under accession number PRJNA1204487. https://www.ncbi.nlm.nih.gov/bioproject/PRJNA1204487.

## References

[B1] Altenbach S. B. Chang H.-C. Rowe M. H. Yu X. B. Simon-Buss A. Seabourn B. W.. (2020). Reducing the immunogenic potential of wheat flour: silencing of alpha gliadin genes in a US wheat cultivar. Front. Plant Sci. 11. doi: 10.3389/fpls.2020.00020 PMC7052357 32161604

[B2] Anderson R. P. Degano P. Godkin A. J. Jewell D. P. Hill A. V. (2000). *In vivo* antigen challenge in celiac disease identifies a single transglutaminase-modified peptide as the dominant A-gliadin T-cell epitope. Nat. Med. 6, 337–342. doi: 10.1038/73200 10700238

[B3] Anderson O. Greene F. (1997). The α-gliadin gene family. II. DNA and protein sequence variation, subfamily structure, and origins of pseudogenes: II. DNA and protein sequence variation, subfamily structure, and origins of pseudogenes. Theor. Appl. Genet. 95, 59–65. doi: 10.1007/s001220050532

[B4] Andrews S. (2010). “FastQC: a quality control tool for high throughput sequence data” (United Kingdom: Cambridge).

[B5] Arentz-Hansen H. Körner R. Molberg Ø. Quarsten H. Vader W. Kooy Y. M.. (2000). The intestinal T cell response to α-gliadin in adult celiac disease is focused on a single deamidated glutamine targeted by tissue transglutaminase. J. Exp. Med. 191, 603–612. doi: 10.1084/jem.191.4.603 10684852 PMC2195837

[B6] Bolger A. M. Lohse M. Usadel B. (2014). Trimmomatic: a flexible trimmer for Illumina sequence data. Bioinformatics 30, 2114–2120. doi: 10.1093/bioinformatics/btu170 24695404 PMC4103590

[B7] Catassi C. Gatti S. Fasano A. (2014). The new epidemiology of celiac disease. J. Pediatr. Gastroenterol. Nutr. 59, S7–S9. doi: 10.1097/01.mpg.0000450393.23156.59 24979197

[B8] Chen C. Wu Y. Li J. Wang X. Zeng Z. Xu J.. (2023). TBtools-II: A “one for all, all for one” bioinformatics platform for biological big-data mining. Mol. Plant 16, 1733–1742. doi: 10.1016/j.molp.2023.09.010 37740491

[B9] Chomczynski P. Sacchi N. (2006). The single-step method of RNA isolation by acid guanidinium thiocyanate–phenol–chloroform extraction: twenty-something years on. Nat. Protoc. 1, 581–585. doi: 10.1038/nprot.2006.83 17406285

[B10] Crooks G. E. Hon G. Chandonia J.-M. Brenner S. E. (2004). WebLogo: a sequence logo generator. Genome Res. 14, 1188–1190. doi: 10.1101/gr.849004 15173120 PMC419797

[B11] El Khoury D. Balfour-Ducharme S. Joye I. J. (2018). A review on the gluten-free diet: Technological and nutritional challenges. Nutrients 10, 1410. doi: 10.3390/nu10101410 30279384 PMC6213115

[B12] Fallang L.-E. Bergseng E. Hotta K. Berg-Larsen A. Kim C.-Y. Sollid L. M. (2009). Differences in the risk of celiac disease associated with HLA-DQ2. 5 or HLA-DQ2. 2 are related to sustained gluten antigen presentation. Nat. Immunol. 10, 1096–1101. doi: 10.1038/ni.1780 19718029

[B13] FAO (2022). FAOSTAT. Available online at: https://www.fao.org/faostat/ (Accessed August 10, 2022).

[B14] Gil-Humanes J. Pistón F. Altamirano-Fortoul R. Real A. Comino I. Sousa C.. (2014). Reduced-gliadin wheat bread: an alternative to the gluten-free diet for consumers suffering gluten-related pathologies. PloS One 9, e90898. doi: 10.1371/journal.pone.0090898 24621595 PMC3951262

[B15] Gil-Humanes J. Pistón F. Tollefsen S. Sollid L. M. Barro F. (2010). Effective shutdown in the expression of celiac disease-related wheat gliadin T-cell epitopes by RNA interference. Proc. Natl. Acad. Sci. 107, 17023–17028. doi: 10.1073/pnas.1007773107 20829492 PMC2947919

[B16] Gouy M. Guindon S. Gascuel O. (2010). SeaView version 4: a multiplatform graphical user interface for sequence alignment and phylogenetic tree building. Mol. Biol. Evol. 27, 221–224. doi: 10.1093/molbev/msp259 19854763

[B17] Halstead-Nussloch G. Tanaka T. Copetti D. Paape T. Kobayashi F. Hatakeyama M.. (2021). Multiple wheat genomes reveal novel gli-2 sublocus location and variation of celiac disease epitopes in duplicated α-gliadin genes. Front. Plant Sci. 12. doi: 10.3389/fpls.2021.715985 PMC8446623 34539709

[B18] Harberd N. Bartels D. Thompson R. (1985). Analysis of the gliadin multigene loci in bread wheat using nullisomic-tetrasomic lines. Mol. Gen. Genet. MGG 198, 234–242. doi: 10.1007/bf00383001

[B19] Huo N. Zhu T. Altenbach S. Dong L. Wang Y. Mohr T.. (2018). Dynamic evolution of α-gliadin prolamin gene family in homeologous genomes of hexaploid wheat. Sci. Rep. 8, 5181. doi: 10.1038/s41598-018-23570-5 29581476 PMC5980091

[B20] Jouanin A. Gilissen L. J. Boyd L. A. Cockram J. Leigh F. J. Wallington E. J.. (2018). Food processing and breeding strategies for coeliac-safe and healthy wheat products. Food Res. Int. 110, 11–21. doi: 10.1016/j.foodres.2017.04.025 30029701

[B21] Jouanin A. Schaart J. G. Boyd L. A. Cockram J. Leigh F. J. Bates R.. (2019). Outlook for coeliac disease patients: towards bread wheat with hypoimmunogenic gluten by gene editing of α-and γ-gliadin gene families. BMC Plant Biol. 19, 1–16. doi: 10.1186/s12870-019-1889-5 31370789 PMC6670228

[B22] Khan K. Hamada A. Patek J. (1985). Polyacrylamide gel electrophoresis for wheat variety identification: Effect of variables on gel properties. Cereal Chem. 62, 310–313.

[B23] Kishii M. (2019). An update of recent use of Aegilops species in wheat breeding. Front. Plant Sci. 10. doi: 10.3389/fpls.2019.00585 PMC6521781 31143197

[B24] Kolde R. (2019). Pheatmap: pretty heatmaps. R Package version 1, 726. doi: 10.32614/cran.package.pheatmap

[B25] Kumar A. Kapoor P. Chunduri V. Sharma S. Garg M. (2019). Potential of Aegilops sp. for improvement of grain processing and nutritional quality in wheat (Triticum aestivum). Front. Plant Sci. 10. doi: 10.3389/fpls.2019.00308 PMC6431632 30936886

[B26] Li W. Godzik A. (2006). Cd-hit: a fast program for clustering and comparing large sets of protein or nucleotide sequences. Bioinformatics 22, 1658–1659. doi: 10.1093/bioinformatics/btl158 16731699

[B27] Ludvigsson J. F. Leffler D. A. Bai J. C. Biagi F. Fasano A. Green P. H.. (2013). The Oslo definitions for coeliac disease and related terms. Gut 62, 43–52. doi: 10.1136/gutjnl-2011-301346 22345659 PMC3440559

[B28] Magoč T. Salzberg S. L. (2011). FLASH: fast length adjustment of short reads to improve genome assemblies. Bioinformatics 27, 2957–2963. doi: 10.1093/bioinformatics/btr507 21903629 PMC3198573

[B29] Maiuri L. Ciacci C. Ricciardelli I. Vacca L. Raia V. Auricchio S.. (2003). Association between innate response to gliadin and activation of pathogenic T cells in coeliac disease. Lancet 362, 30–37. doi: 10.1016/s0140-6736(03)13803-2 12853196

[B30] Marín-Sanz M. Barro F. Sánchez-León S. (2023). Unraveling the celiac disease-related immunogenic complexes in a set of wheat and tritordeum genotypes: implications for low-gluten precision breeding in cereal crops. Front. Plant Sci. 14. doi: 10.3389/fpls.2023.1171882 PMC10210591 37251754

[B31] Mirzaghaderi G. Abdolmalaki Z. Ebrahimzadegan R. Bahmani F. Orooji F. Majdi M.. (2020). Production of synthetic wheat lines to exploit the genetic diversity of emmer wheat and D genome containing Aegilops species in wheat breeding. Sci. Rep. 10, 19698. doi: 10.1038/s41598-020-76475-7 33184344 PMC7661528

[B32] Mitea C. Salentijn E. M. van Veelen P. Goryunova S. V. van der Meer I. M. van den Broeck H. C.. (2010). A universal approach to eliminate antigenic properties of alpha-gliadin peptides in celiac disease. PloS One 5, e15637. doi: 10.1371/journal.pone.0015637 21179575 PMC3002971

[B33] Ozuna C. V. Iehisa J. C. Giménez M. J. Alvarez J. B. Sousa C. Barro F. (2015). Diversification of the celiac disease α-gliadin complex in wheat: a 33-mer peptide with six overlapping epitopes, evolved following polyploidization. Plant J. 82, 794–805. doi: 10.1111/tpj.12851 25864460

[B34] Qiao S.-W. Bergseng E. Molberg Ø. Xia J. Fleckenstein B. Khosla C.. (2004). Antigen presentation to celiac lesion-derived T cells of a 33-mer gliadin peptide naturally formed by gastrointestinal digestion. J. Immunol. 173, 1757–1762. doi: 10.4049/jimmunol.173.3.1757 15265905

[B35] Salentijn E. M. Esselink D. G. Goryunova S. V. van der Meer I. M. Gilissen L. J. Smulders M. J. (2013). Quantitative and qualitative differences in celiac disease epitopes among durum wheat varieties identified through deep RNA-amplicon sequencing. BMC Genomics 14, 1–16. doi: 10.1186/1471-2164-14-905 24354426 PMC3890609

[B36] Salentijn E. M. Goryunova S. V. Bas N. van der Meer I. M. van den Broeck H. C. Bastien T.. (2009). Tetraploid and hexaploid wheat varieties reveal large differences in expression of alpha-gliadins from homoeologous Gli-2 loci. BMC Genomics 10, 1–14. doi: 10.1186/1471-2164-10-48 19171027 PMC2636828

[B37] Schaart J. G. Salentijn E. M. Goryunova S. V. Chidzanga C. Esselink D. G. Gosman N.. (2021). Exploring the alpha-gliadin locus: the 33-mer peptide with six overlapping coeliac disease epitopes in Triticum aestivum is derived from a subgroup of Aegilops tauschii. Plant J. 106, 86–94. doi: 10.1111/tpj.15147 33369792 PMC8248119

[B38] Shan L. Molberg Ø. Parrot I. Hausch F. Filiz F. Gray G. M.. (2002). Structural basis for gluten intolerance in celiac sprue. Science 297, 2275–2279. doi: 10.1126/science.1074129 12351792

[B39] Shewry P. R. (2009). Wheat. J. Exp. Bot. 60, 1537–1553. doi: 10.1093/jxb/erp058 19386614

[B40] Shewry P. (2019). What is gluten—why is it special? Front. Nutr. 6. doi: 10.3389/fnut.2019.00101 PMC6625226 31334243

[B41] Shewry P. R. Halford N. G. Belton P. S. Tatham A. S. (2002). The structure and properties of gluten: an elastic protein from wheat grain. Philos. Trans. R. Soc. London. Ser. B: Biol. Sci. 357, 133–142. doi: 10.1098/rstb.2001.1024 11911770 PMC1692935

[B42] Shewry P. R. Hey S. J. (2015). The contribution of wheat to human diet and health. Food Energy Secur. 4, 178–202. doi: 10.1002/fes3.64 27610232 PMC4998136

[B43] Singh P. Arora A. Strand T. A. Leffler D. A. Catassi C. Green P. H.. (2018). Global prevalence of celiac disease: systematic review and meta-analysis. Clin. Gastroenterol. Hepatol. 16, 823–836. doi: 10.2139/ssrn.4824540 29551598

[B44] Sollid L. M. (2002). Coeliac disease: dissecting a complex inflammatory disorder. Nat. Rev. Immunol. 2, 647–655. doi: 10.1038/nri885 12209133

[B45] Sollid L. M. Qiao S.-W. Anderson R. P. Gianfrani C. Koning F. (2012). Nomenclature and listing of celiac disease relevant gluten T-cell epitopes restricted by HLA-DQ molecules. Immunogenetics 64, 455–460. doi: 10.1007/s00251-012-0599-z 22322673 PMC3349865

[B46] Sollid L. M. Tye-Din J. A. Qiao S.-W. Anderson R. P. Gianfrani C. Koning F. (2020). Update 2020: nomenclature and listing of celiac disease–relevant gluten epitopes recognized by CD4+ T cells. Immunogenetics 72, 85–88. doi: 10.1007/s00251-019-01141-w 31735991

[B47] Spaenij–Dekking L. Kooy–Winkelaar Y. van Veelen P. Drijfhout J. W. Jonker H. van Soest L.. (2005). Natural variation in toxicity of wheat: potential for selection of nontoxic varieties for celiac disease patients. Gastroenterology 129, 797–806. doi: 10.1053/j.gastro.2005.06.017 16143119

[B48] Tamura K. Stecher G. Kumar S. (2021). MEGA11: molecular evolutionary genetics analysis version 11. Mol. Biol. Evol. 38, 3022–3027. doi: 10.1093/molbev/msab120 33892491 PMC8233496

[B49] Tye-Din J. A. Stewart J. A. Dromey J. A. Beissbarth T. van Heel D. A. Tatham A.. (2010). Comprehensive, quantitative mapping of T cell epitopes in gluten in celiac disease. Sci. Trans. Med. 2, 41ra51–41ra51. doi: 10.1126/scitranslmed.3001012 20650871

[B50] van Herpen T. W. Goryunova S. V. van der Schoot J. Mitreva M. Salentijn E. Vorst O.. (2006). Alpha-gliadin genes from the A, B, and D genomes of wheat contain different sets of celiac disease epitopes. BMC Genomics 7, 1–13. doi: 10.1186/1471-2164-7-1 16403227 PMC1368968

[B51] Vriz R. Moreno F. J. Koning F. Fernandez A. (2021). Ranking of immunodominant epitopes in celiac disease: Identification of reliable parameters for the safety assessment of innovative food proteins. Food Chem. Toxicol. 157, 112584. doi: 10.1016/j.fct.2021.112584 34582965

[B52] Wang D.-W. Li D. Wang J. Zhao Y. Wang Z. Yue G.. (2017). Genome-wide analysis of complex wheat gliadins, the dominant carriers of celiac disease epitopes. Sci. Rep. 7, 1–14. doi: 10.1038/srep44609 28300172 PMC5353739

[B53] Watry H. Zerkle A. Laudencia-Chingcuanco D. (2020). Modified acid-PAGE method for rapid screening and phenotyping of wheat gliadin mutant lines. MethodsX 7, 100858. doi: 10.1016/j.mex.2020.100858 32322542 PMC7163331

