# Supplementary material for: Variations in the composition and frequency of celiac disease epitopes among synthetic wheat lines
Source: Front Plant Sci. 2025 Jan 27;15:1517821. doi: 10.3389/fpls.2024.1517821 (PMC11807966; doi:10.3389/fpls.2024.1517821)
Supplement: Supplementary file 2 [file SupplementaryFile1.docx]

Supplementary Material

# Supplementary Figures


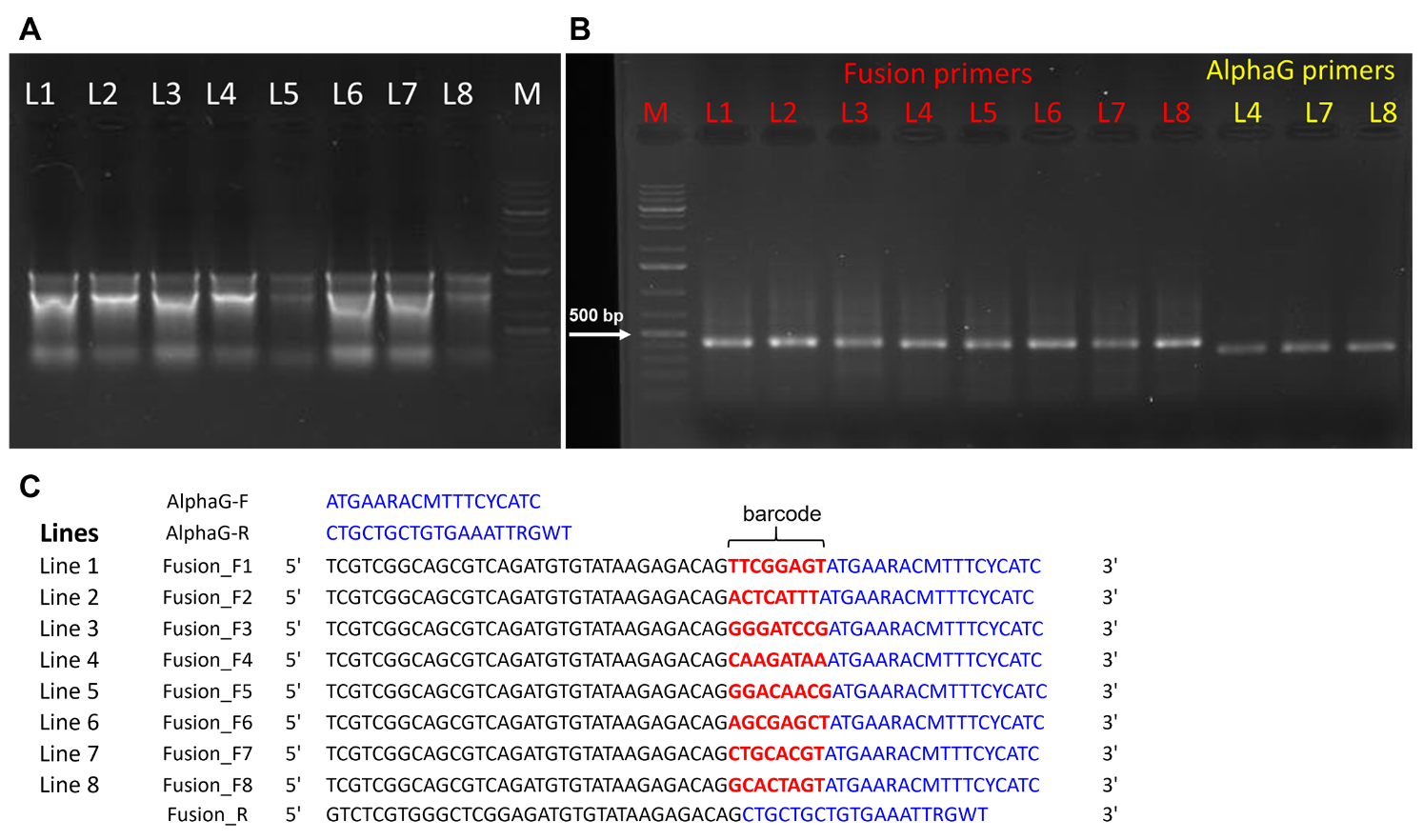


**Supplementary Figure 1.** Agarose gel electrophoresis and primers used in this study. **(A)** Agarose gel electrophoresis of RNA extracted from wheat seeds during grain filling at 21 days post-anthesis. **(B)** Electrophoresis bands of alpha-gliadin PCR products amplified from cDNA using alpha-gliadin primers (yellow wells; First PCR) and fusion primers (red wells; Second PCR using the First PCR product as template). **(C)** Primers used in this research. The original alpha-gliadin primers are shown in blue. Each forward fusion primer contains the adapter sequence for Illumina preparation library (in black), barcode sequence specific to each synthetic line (in red), and the original alpha-gliadin forward primer (in blue). The reverse fusion primer contains the Illumina adapter sequence and original alpha-gliadin reverse primer. Different synthetic wheat lines are indicated as L1 to L8 as described in Table 1.


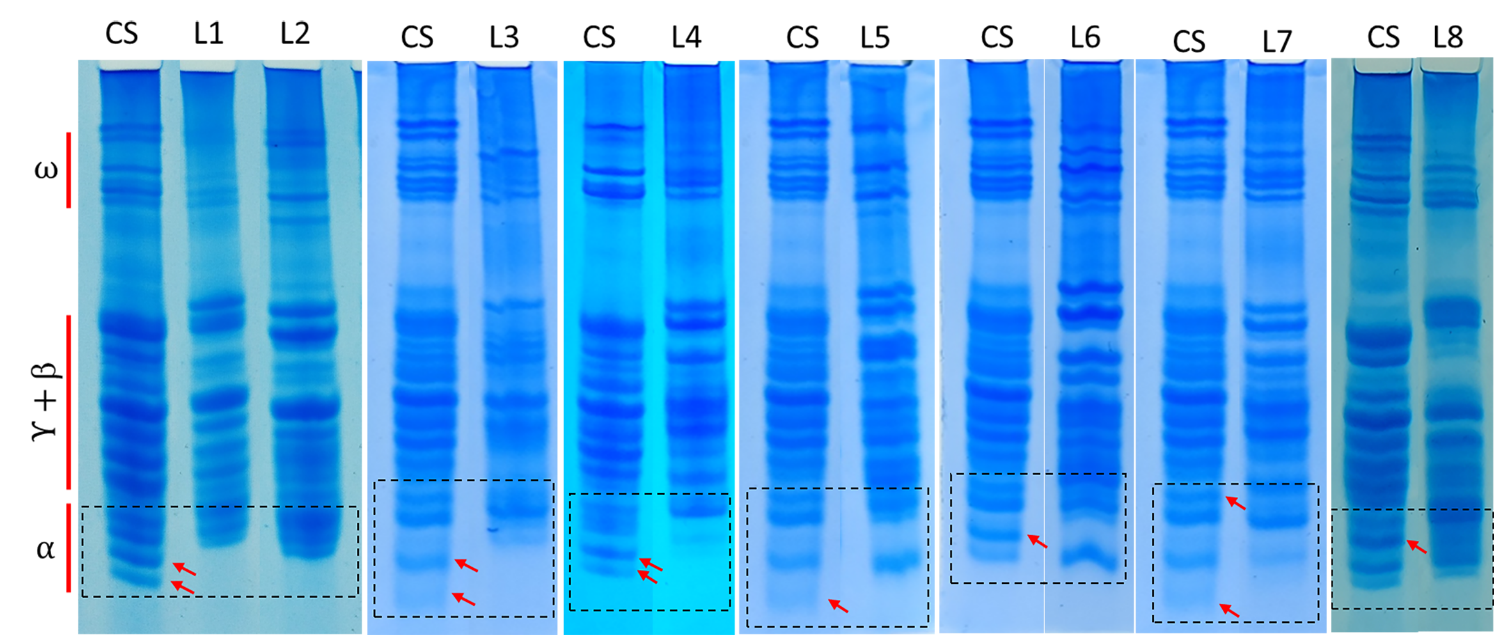


**Supplementary Figure 2.** Acid-PAGE patterns of gliadins among different synthetic wheats. The lane “CS’ shows banding pattern of alpha gliadin in Chinese spring wheat (*Triticum aestivum* L.). Details about different synthetic wheat lines (L1 to L8) including their pedigree are presented in Table 1. The position of alpha-gliadin bands is represented inside the dashed shapes. Arrows indicate the position of bands missing in the corresponding synthetic line.
